# Hypovitaminosis D in Obese and Overweight Schoolchildren

**DOI:** 10.4008/jcrpe.v1i2.43

**Published:** 2008-11-05

**Authors:** Filiz Mine Çizmecioğlu, Nilay Etiler, Uzay Görmüş, Onur Hamzaoğlu, Şükrü Hatun

**Affiliations:** 1 Division of Endocrinology and Diabetes, Department of Pediatrics, Kocaeli University, Faculty of Medicine, Kocaeli, Turkey; 2 Department of Public Health, Kocaeli University, Faculty of Medicine, Kocaeli, Turkey; 3 Department of Molecular Medicine, İstanbul University, İstanbul, Turkey; +90−262−303 72 28+90−262−303 80 03filizcizmeci@gmail.comDivision of Endocrinology and Diabetes, Department of Pediatrics, Kocaeli University, Faculty of Medicine Kocaeli, Turkey 41380

**Keywords:** adolescents, obesity, Vitamin D, hypovitaminosis D, schoolchildren

## Abstract

**Aim**: To determine the prevalence of vitamin D hypovitaminosis among obese and overweight schoolchildren.

**Design**: A cross−sectional population based sample.

**Methods**: In a cross−sectional study, 301 students (177 girls and 124 boys) aged 11−19 years were selected by multistage stratified sampling design. Subjects were classified according to their body mass index as obese, overweight and normal. Serum 25−hydroxyvitamin D (25−OHD), intact parathyroid hormone (iPTH) and alkaline phosphatase (ALP) were measured in late winter months. Vitamin D deficiency was defined as a 25−OHD 20 ng/ml.

**Results**: The prevalence of hypovitaminosis D was found as 65% in all students. Vitamin D deficiency was found in 12% and insufficiency in 53% of all students. Vitamin D deficiency in female students was about two times more common than in males. In obese and overweight schoolchildren with hypovitaminosis D, serum 25−OHD levels decreased as BMI increased. There were no correlations between serum 25−OHD and ALP and iPTH levels.

**Conclusion**: Vitamin D deficiency and insufficiency are common in obese and overweight schoolchildren, especially in girls. Obesity could be a risk factor in terms of hypovitaminosis D in adolescents. Vitamin D supplementation should be administered particularly to adolescent girls.

**Conflict of interest:**None declared.

## INTRODUCTION

Recently clinical and subclinical vitamin D deficiency has, once again, become a current issue. High rates of subclinical vitamin D deficiency have been reported among some risk groups such as immigrants in Europe from the Middle East and Asia, African Americans and adolescents from various countries.(1, 2, 3, 4, 5, 6) Vitamin D insufficiency among healthy adolescents was reported as 78% in France, 65% in Finland, 52% in Beirut, 42.5% in Beijing, 42% in Boston, 47% in Greece, 46.2% in Iran and 29% in Switzerland.(7, 8, 9, 10, 11, 12, 13, 14, 15, 16) Similarly in Turkey, hypovitaminosis D rate is 59.4% among adolescents in Izmir, which is a sunny seaside city.(17) Previously we had reported high rates of subclinical vitamin D deficiency (65%) in adolescent girls who wear concealing clothing in the Kocaeli region.(18)

Inadequate vitamin D intake has been associated with obesity in young adults.(19) Recent limited studies have pointed out a relation between obesity and vitamin D hypovitaminosis which are frequent in adolescence because of changing life styles.(20)

In this study we aimed to determine the prevalence of vitamin D hypovitaminosis in both sexes among schoolchildren, many of whom were obese and overweight in our highly industrialized city.

## METHODS

**Subjects**

This cross−sectional study was performed in Kocaeli, a highly industrialized region located in northwestern Turkey at a latitude of 30°E and 40°N in the late winter months. Participants for this study were recruited from the study samples which consisted of secondary and highschoolchildren, aged 11−18 years, using a multistage, stratified sampling design for the epidemiological study to determine metabolic syndrome prevalence. A total of 301 schoolchildren from 3 different towns (İzmit, Gebze and Kandıra) were included in the study. The first two of these towns are highly industrialized areas and third is a non−industrialized seaside town. For sample selection, the schools were stratified and weighted first as urban vs rural area and next as state owned vs private school. After obtaining each stratum’s weights, the schools and the students were selected using random numbers from the students’ list. At the first stage of the study, anthropometric measurements of 2491 subjects participating in the research were performed in the schools after obtaining verbal informed consent from the children. At the second stage, participants whose body mass index (BMI, calculated as kg/m^2^) was ≥85^th^ centile were invited to the hospital for further investigation. CDC reference values were used for assessment of overweight and obesity.(21) Of these 301 children and adolescents who were included in the study, 102 were obese (34%), 145 were overweight (48%). BMI values were withinnormal percentile ranges in 54 (18%) whohad lost weight and returned to normal BMI when the blood samples were collected for further investigation. BMI standard deviation score (SDS) and centiles were obtained according to CDC reference value.(21)

Subjects were evaluated according to pubertal stages. Tanner stage I was defined as pre−puberty, Tanner stage II−IV as midpuberty and Tanner stage V as post−puberty.

Children with any systemic disease or using any medications or supplements known to affect skeletal metabolism were excluded from the study. Written informed consent was also obtained from all parents and subjects at the second stage of the study. This study was approved by the Ethics Committee of Kocaeli University. Permission for the study was granted by the Directorate of National Education of Kocaeli.

**Laboratory tests**

After an overnight fast morning blood samples were taken from the participants for serum 25−hydroxyvitamin D (25−OHD), intact parathyroid hormone (iPTH) and alkaline phosphatase (ALP) measurements. Vitamin D deficiency was defined as a 25− OHD blood level of <10 ng/ml, vitamin D insufficiency as levels of 25−OHD between 10 and 20 ng/ml, and a normal vitamin D level as >20 ng/ml.(22, 23) The subjects were divided into 3 categories (vitamin D deficiency, group A; vitamin D insufficiency, group B; normal or vitamin D competent, group C), according to their serum 25−OHD concentrations.

A competitive protein binding assay was used to measure 25−OH Vit D levels (Vita D EIA kit, Immundiagnostic, Bensheim, Germany). The normal range for 25−OHD was stated as 11 to 70 ng/mL and intra−and interassay coefficients of variation (CVs) were 10.7% and 13.2% respectively. Serum iPTH was measured by using chemiluminescence with an Immulite One analyzer. The reference range suggested by this method is 12−65 pg/ml, and total coefficients of variation are 2.8%, and 3.4% respectively. Serum levels of ALP were measured using original assays by Aeroset autoanalyser equipment.

**Statistical analysis**

The data were analyzed with Statistical Package for the Social Sciences (SPSS Inc., Chicago) Version 11.5. Two sample independent group t−test was performed to compare some characteristics and 25−OHD categories between boys and girls. The chi−square test was used to assess frequency differences between 25−OHD categories. The relationships between 25− OHD and some variables (BMI, ALP, PTH) were evaluated using a Pearson correlation coefficient. Significance was accepted at a p<0.05.

## RESULTS

**Characteristics of the study groups**

A total of 301 schoolchildren and adolescents were studied. The mean age (range) of the students was 14.2 ± 1.8 (11.0−18.7). 59% were female (n=177) and 41% (n=124) male, 67% were (n=201) post−pubertal, 29% (n=88) were mid−pubertal (Tanner stage between IIIV) and 4% were (n=12) pre−pubertal. Fifty nine percent of the children (n=177) were secondary school students and 41% (n=124) were in highschool. Their anthropometric characteristics are shown in Table 1.

**Prevalence of hypovitaminosis D**

Serum levels of 25−OHD in the total group ranged between 2.8 and 72 ng/ml. The mean level of 25−OHD was significantly lower in girls compared to boys (M; 20.7±9.5, F; 16.4±8.8) (p<0.01). Prevalence of vitamin D deficiency was 12% and insufficiency was 53% in the total group. The proportion of children with vitamin D levels below normal was 65%. There were significant differences in vitamin D levels between the two sexes (p<0.001). Prevalence of vitamin D deficiency in female students, a seen in Table 2, was about two times more common than in males.

Vitamin D status was assessed according to its relationship to obesity and also to place of residence.

**Figure 1:**
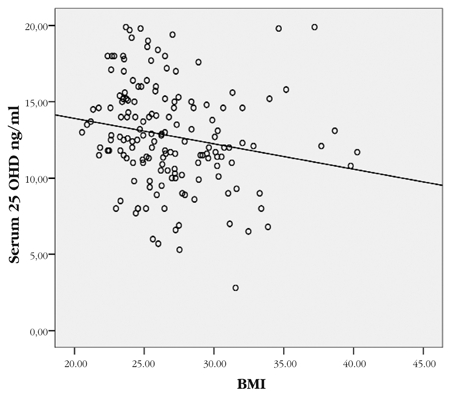
The correlation between serum 25−OHD level and BMI in obese and overweight subjects with vitamin D levels <20 ng/ml. The 25−OHD levels correlated negatively with BMI (r: − 0.186, p< 0.01).

Vitamin D levels and obesity: Mean BMI of the total group was 26.0 ± 3.8 ng/ml (19.3 − 40.3) and mean (range) of BMI SDS was 1.5 ± 0.4 (range 0.3−2.6). Although the girls appeared to have higher BMI values than the boys, there was no statistically significant difference between their BMI SDS values. There was also no relation between obesity status and vitamin D categories (Table 3). However, we found a negative correlation between serum vitamin D level and BMI in obese and overweight subjects whose vitamin D level was below 20 ng/ml (r: – 0.186 p<0.01) (Figure 1).

Vitamin D levels and place of residence: While mean vitamin D level in Kandıra was 22.2±5 ng/ml (12.4−27.4), it was 19.0±11.7 (2.8−11.7) in İzmit and 17.1±6.6 ng/ml (5.7− 40) in Gebze (p=0.078). The prevalence of vitamin D deficiency and insufficiency were low in Kandıra (p<0.05) (Table 4).

There were no relationships between serum 25 OHD and iPTH or ALP levels (r:− 0.074, p=0.202, r: 0.078, p=0.175, respectively).

**Table 1:**
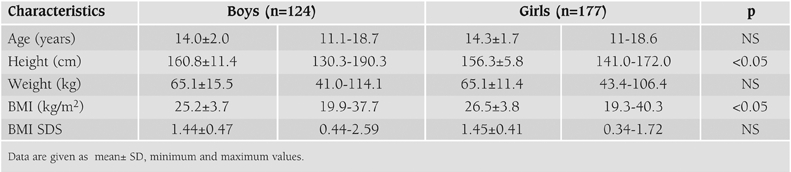
Anthropometric characteristics of the subjects by sex

**Table 2:**
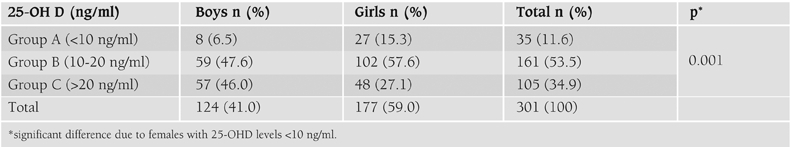
Vitamin D status of subjects by sex

**Table 3:**
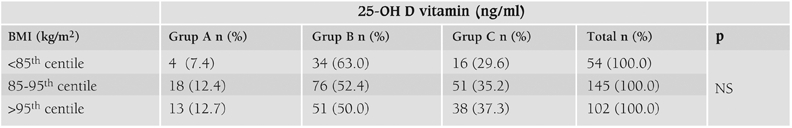
Vitamin D status by obesity status

**Table 4:**
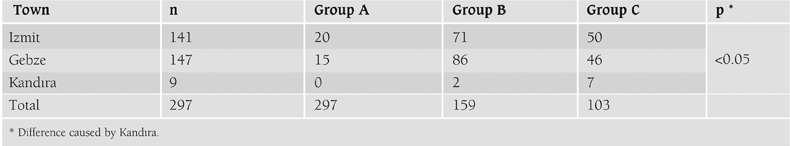
Vitamin D status according by place of residence

## DISCUSSION

In consistency with previous reports, vitamin D deficiency among adolescents in our country is a common problem.17,18 When we use the recently proposed cut−off values for optimal vitamin D level (normal >20 ng/ml, vitamin D deficiency <10 ng/ml and 20−30 ng/ml, vitamin D insufficiency),(24, 26) only 6% of subjects had normal vitamin D concentrations, 29% were vitamin D insufficient and 65% were vitamin D deficient. The main importance of this issue is whether this condition will have any clinical impact on human health at long term. While studies in western countries reported no difference between sexes regarding prevalence of hypovitaminosis D, studies in the Middle East demonstrated a significant deficiency in the girls, as in our study.(8, 9, 16, 18) This might be due to differences in life styles but has not been investigated. While all of the schoolgirls in the study of Moussavi et al(15) were veiled, none were veiled in our study. Although we live in a sunny country, high rates of hypovitaminosis D might be associated with air−pollution in highly industrialized regions. In our study while the rate of vitamin D deficiency was high in industrialized towns, it was much lower in Kandıra, a rural seaside town. Although sample size was limited in Kandıra, the absence of vitamin D deficiency and the high mean serum vitamin D levels suggest the role of UV radiation.

There are two main biochemical parameters regarding the negative effects of vitamin D deficiency on the skeleton namely ALP and iPTH. The cut−off point of serum 25− OHD in which the mean serum PTH concentration begins to increase is defined as 20 or 30 ng/ml.(9, 13, 15, 22, 27, 28, 29) When we used a cut−off point of 20 ng/ml and even a cut−off level 10 ng/ml, we were not able to demonstrate secondary hyperparathyroidism, nor a correlation between serum 25−OHD and ALP and iPTH levels. This might be due to the cross−sectional design of the study. We do not know how long these children have been suffering from hypovitaminosis D. For this reason, ALP and PTH concentrations of subjects with hypovitaminosis D should be followed at long term. Another important issue is the treatment approach in subjects with vitamin D deficiency whose ALP and PTH levels are not elevated. Whether pharmacological doses of vitamin D or physiological doses should be administered is not clear.

There are many reasons for the high frequency of vitamin D deficiency which differ according to countries. In our previous study, the most important reason was concealing clothing.(18) In this study we assessed the impact of obesity. In the literature, some reports showed no interaction of BMI and vitamin D, but particularly studies on adults and a few on children demonstrated a relation between obesity and vitamin D deficiency.(9, 14, 19, 20, 30, 31) We did not observe any relation between obesity status and vitamin D categories. However, in obese and overweight schoolchildren with hypovitaminosis D, serum 25−OHD levels decreased as BMI increased. This inverse relationship is in consistency with the hypothesis suggesting that the increased adipose tissue decreases vitamin D bioavailability by sequestration in body fat.(19, 31) However, whether obese children and adolescents require a separate dose of vitamin D supplementation is controversial.(31)

Although the clinical importance of rickets is well known, the effects of subclinical vitamin D deficiency on human health are still under debate. Vitamin D receptor (VDR) is present in the small intestine, bone and in the kidney, which all have major responsibilities for regulating calcium and phosphorous metabolism. It is reported that the skin, brain, gonads, colon, beta−islet cells, prostate, heart, skeletal muscle, monocytes, activated T and B lymphocytes and adipocytes all express the same VDR.(31, 32) Recent studies on VDR in knockout mice revealed that the effects of vitamin D on skeletal health depend exclusively on intestinal Ca absorption.(33) Therefore, the assumption that in various tissues the effect of vitamin D deficiency below the level affecting Ca homeostasis is mediated through VDR, is controversial. However, in many epidemiological studies hypovitaminosis D is associated with chronic diseases and malignancy.(34, 35, 36)

Subclinical vitamin D deficiency not only affects bone health but also has other more serious non−skeletal consequences such as diabetes, hypertension and malignancy. Therefore, in order to prevent the potential chronic diseases in the future, vitamin D supplementation should be administered throughout childhood to both genders, particularly to adolescent girls. Cross−sectional design and also the limited number of students from the non−industrialized region was the limitation of this study. Further longitudinal studies are necessary to define the dose and duration of vitamin D supplementation in childhood considering the confounding factors that might cause hypovitaminosis D.

## ACKNOWLEDGEMENT

We would like to thank Prof. Dr. Nezih Hekimi, Dr. Dilek Erdonmez, Dr. Cavit Işık Yavuz due to contributions about statistical analysis, data collection and vitamin D assay, and we are grateful to intern doctors, nurses and staff who worked during the 1^st^ and 2^nd^ steps of the study. Finally, we express our gratitude to the children and their families for making this study possible.
